# Human Nasal Challenge with *Streptococcus pneumoniae* Is Immunising in the Absence of Carriage

**DOI:** 10.1371/journal.ppat.1002622

**Published:** 2012-04-05

**Authors:** Adam K. A. Wright, Daniela M. Ferreira, Jenna F. Gritzfeld, Angela D. Wright, Kathryn Armitage, Kondwani C. Jambo, Emily Bate, Sherouk El Batrawy, Andrea Collins, Stephen B. Gordon

**Affiliations:** 1 Respiratory Infection Group, Liverpool School of Tropical Medicine, Liverpool, United Kingdom; 2 NIHR Biomedical Research Centre in Microbial Diseases, Royal Liverpool and Broadgreen University Hospitals NHS Trust, Liverpool, United Kingdom; 3 Malawi-Liverpool-Wellcome Trust Clinical Research Programme, Chichiri, Blantyre, Malawi; Children's Hospital Boston, United States of America

## Abstract

Infectious challenge of the human nasal mucosa elicits immune responses that determine the fate of the host-bacterial interaction; leading either to clearance, colonisation and/or disease. Persistent antigenic exposure from pneumococcal colonisation can induce both humoral and cellular defences that are protective against carriage and disease. We challenged healthy adults intra-nasally with live 23F or 6B *Streptococcus pneumoniae* in two sequential cohorts and collected nasal wash, bronchoalveolar lavage (BAL) and blood before and 6 weeks after challenge. We hypothesised that both cohorts would successfully become colonised but this did not occur except for one volunteer. The effect of bacterial challenge without colonisation in healthy adults has not been previously assessed. We measured the antigen-specific humoral and cellular immune responses in challenged but not colonised volunteers by ELISA and Flow Cytometry. Antigen-specific responses were seen in each compartment both before and after bacterial challenge for both cohorts. Antigen-specific IgG and IgA levels were significantly elevated in nasal wash 6 weeks after challenge compared to baseline. Immunoglobulin responses to pneumococci were directed towards various protein targets but not capsular polysaccharide. 23F but not 6B challenge elevated IgG anti-PspA in BAL. Serum immunoglobulins did not increase in response to challenge. In neither challenge cohort was there any alteration in the frequencies of TNF, IL-17 or IFNγ producing CD4 T cells before or after challenge in BAL or blood. We show that simple, low dose mucosal exposure with pneumococci may immunise mucosal surfaces by augmenting anti-protein immunoglobulin responses; but not capsular or cellular responses. We hypothesise that mucosal exposure alone may not replicate the systemic immunising effect of experimental or natural carriage in humans.

## Author Summary

Exposure to respiratory pathogens such as *Streptococcus pneumoniae* (pneumococcus) is a frequent event that can result in immediate clearance, nasal colonisation or disease for the host. Human and mouse studies have shown that natural colonisation is an immunising event. Colonisation is prevalent in children but rare in human adults (<10%), suggesting that despite high pneumococcal exposure adult mucosal defences are sufficient to prevent colonisation. We exposed healthy adults to pneumococci in the nose in order to achieve colonisation and mimic a natural colonisation event. In most volunteers, however, we were not able to obtain colonisation using this protocol. In exposed but not colonised volunteers we measured antibody and cellular responses in nose, lung and blood samples. The mucosal defences elicited during acute pneumococcal exposure are poorly described but these data will shed light on the mechanisms that prevent colonisation in healthy adults and inform future vaccine design. Live bacterial exposure increases specific antibody and innate responses at mucosal surfaces such as the nose and lung. Systemic responses were not increased. These data suggest that acute bacterial exposure per se augments mucosal but not systemic defences. Natural or experimental colonisation may be required for systemic immunisation.

## Introduction

The human nasal mucosa forms the first line of defence against challenge with inhaled bacteria, viruses and non-infectious particles. The highly vascularised mucosa is an attractive niche which permits a large and diverse community of bacterial species to asymptomatically colonise the upper respiratory tract [1]. Invasion of the mucosa by colonising flora is prevented by innate defence mechanisms supported by an interacting sub-mucosal network of antigen presenting cells (macrophages and dendritic cells) [2] with effector T and B lymphocytes [3], [4]. The balance between mucosal immune responses and the expression and immunogenicity of bacterial virulence factors influence both colonisation success and occurrence of invasive disease. *Streptococcus pneumoniae* (pneumococcus) is a common nasal coloniser capable of causing life threatening human disease worldwide [5]. Capsular polysaccharide is a critical virulence factor but anti-capsular antibodies alone do not account for the age related drop observed in the rates of colonisation [6] or invasive disease [7]. An array of additional virulence factors including pneumococcal surface proteins A (PspA) and C (PspC) that mediate attachment to epithelial cells and the pore forming toxin pneumolysin have been shown to be critical for bacterial evasion of host defence [8]. These pneumococcal proteins are immunogenic during colonisation and/or disease [9] and are therefore of interest as vaccine candidates. Mucosal vaccination with non-encapsulated whole bacterial cells [10] or pneumococcal proteins [11], [12] are attractive strategies to elicit capsule independent immunity against pneumococcal colonisation and/or disease.

Pneumococcal carriage is common in infants, particularly among crowded, impoverished communities worldwide where carriage rates range between 20 and 95% [5]. Infants serve as the source of transmission to other children and adults [13] but carriage rates decrease with increasing age as specific immunity develops [14], [15]. The pneumococcal specific immune responses that develop during carriage and protect against subsequent colonisation have been documented in mice and involve both antigen specific T cells and specific antibody [14], [16]–[19]. There are reports in humans correlating T cell [20] and antibody [21], [22] responses to carriage. Pneumococcal carriage in infants [23], [24] and adults [22], [25] in the absence of clinical invasive infection has been associated with increases in the serum levels of immunoglobulin against pneumococcal proteins and capsular polysaccharide. Responses to pneumococcal proteins and to some extent capsular polysaccharides [26] depend on support from antigen-specific T cells to generate optimum levels of specific immunoglobulin. Carriage is known to be an immunising event [25] but it is likely that exposure without carriage also boosts levels of existing immunity in healthy adults [27]. The end result is that in European adults in contrast to infants, the rate of pneumococcal carriage is very low despite frequent exposures.

We measured mucosal humoral and cellular immune responses before and after intra-nasal live pneumococcal challenge in human volunteers. We anticipated that pneumococcal intra-nasal challenge would lead to nasopharyngeal colonisation in our volunteers. Our hypothesis was therefore that challenge would lead to colonisation which would in turn elicit a humoral and cellular mucosal response. Our hypothesis also included the converse argument - that acute pneumococcal exposure (without colonisation) would not elicit a humoral and cellular mucosal response. Systemic immunisation requires efficient targeting and retention of antigen at inductive sites such as M cells or dendritic cells for priming in naso-associated lymphoid tissue (NALT) [28] and we did not know if this would occur or not following an acute exposure to pneumococcus. Our results showed that we did not achieve colonisation with 23F or 6B serotypes in 19 healthy adults. The pneumococcal exposure alone increased mucosal but not systemic pneumococcal specific immunoglobulin responses. Cellular immunity in mucosal and systemic compartments was unaltered by pneumococcal challenge.

## Materials and Methods

### Recruitment and ethics

Healthy adult volunteers were enrolled with written informed consent to a study involving inoculation of either *Streptococcus pneumoniae* type 23F or 6B, the two studies performed consecutively. We recruited non-smoking adults aged between 18 and 60 years with no history or signs of systemic or respiratory disease. Individuals who were already naturally colonised with pneumococcus or had regular contact with at risk individuals, such as young children, were excluded from the study. Ethical approval was obtained from the National Health Service Research Ethics Committee, Sefton, Liverpool (08/H1001/52) and the study was sponsored by the Royal Liverpool and Broadgreen University Hospitals Trust.

### Study design

**Figure 1:**
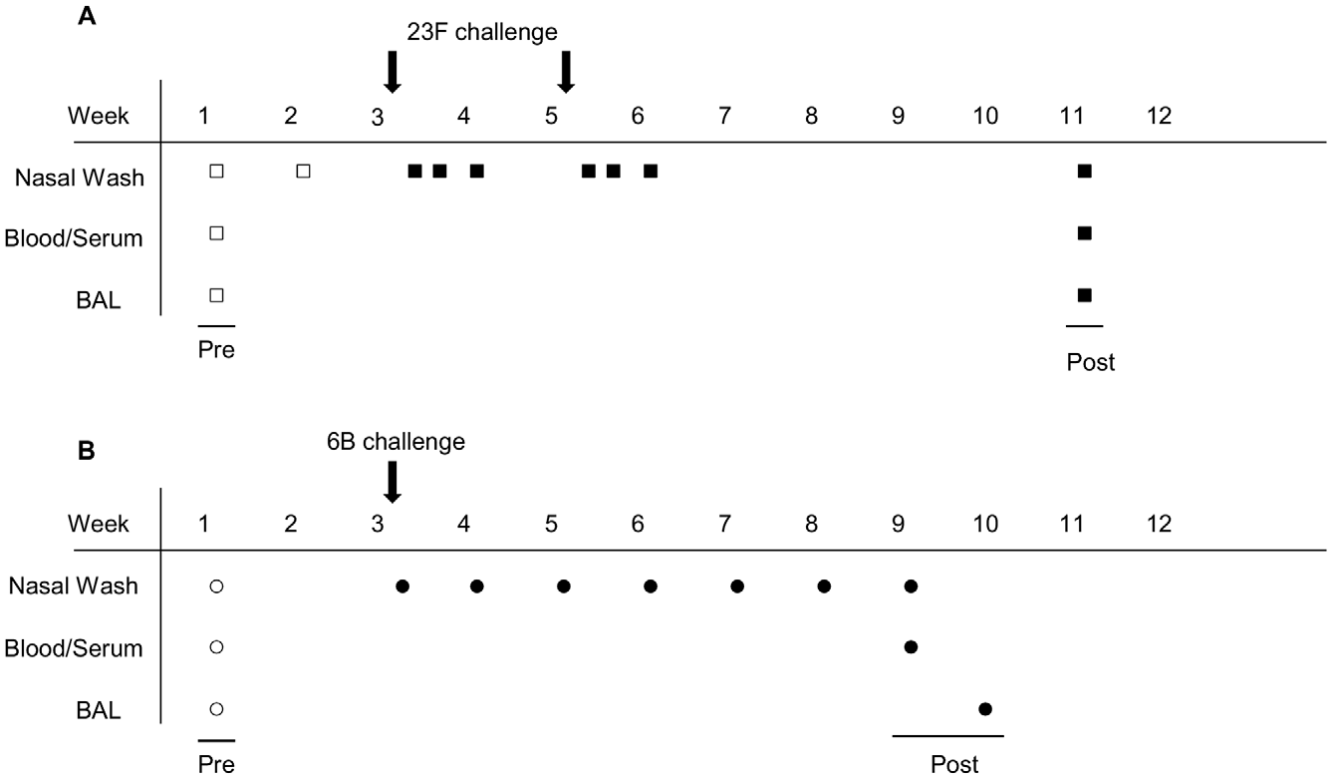
Experimental human intra-nasal 23F and 6B pneumococcal challenge study design. (A) 23F challenge and (B) 6B challenge. NW, blood and BAL were obtained from each volunteer prior to (open symbols) and following (closed symbols) challenge with 23F (Arrows in upper panel *n* = 8) or 6B (Arrow in lower panel *n* = 11).

Samples of blood, serum, nasal wash (NW) and bronchoalveolar lavage (BAL) were obtained before and after bacterial challenge according to two different sampling protocols (Figure 1). Volunteers in our first study were intra-nasally challenged with two doses of 23F (Figure 1A) and in the second study a single dose of 6B (Figure 1B) *S. pneumoniae* was used. In the 23F challenge cohort NW samples were collected 2 weeks and 1 week before inoculation and on days 2, 4, and 7 after each inoculation. In the 6B challenge cohort (Figure 1B) NWs were collected 2 weeks before challenge and 2 and 7 days following inoculation. NWs were then collected once per week for 5 consecutive weeks. A final set of post-challenge samples (blood, serum, NW and BAL) were then collected at 6 weeks.

### 23F and 6B preparation and volunteer inoculation

Clinical isolates of *S. pneumoniae* serotype 23F (P833 a gift of Prof. JN Weiser, University of Pennsylvania) and 6B (BHN418 a gift of Prof. P Hermans, University of Nijmegen) were used for inoculation. Bacterial stocks were grown to mid-log phase in Vegitone broth (Oxoid) [29] in order to avoid volunteer exposure to animal products and stored in 1 ml aliquots of Vegitone with 10% glycerol (Sigma-Aldrich) at −80°C. Serotype confirmation was performed using latex agglutination (Statens Serum Institute, Copenhagen) and was confirmed by an independent reference laboratory (Health Protection Agency, Colindale, UK).

On each day of inoculation two aliquots were thawed, centrifuged and bacterial pellets were washed once before being re-suspended and diluted in sterile 0.9% saline to reach the desired concentration of bacteria. For the 23F cohort 9,000 CFU/naris in 100 µl saline were used for the first inoculation. If colonisation was not established following this inoculation we performed a second inoculation with 20,000 CFU/naris in 100 µl saline. For the 6B cohort 40,000CFU/100 µl/naris was administered. Volunteers tilted their head slightly back and 100 µl of bacterial suspension was dropped onto each naris using a P200 pipette taking care not to touch the mucosa. Serial dilutions of the original inocula were plated onto blood agar for dose confirmation (Table 1).

**Table 1:** 23F and 6B cohort study details.

	23F	6B
Total number of volunteers	8	11
Gender (M∶F)	4∶4	4∶7
Age (mean±SD)	31±16	25±6
1^st^ challenge dose (per naris) (mean±SD)	8,791±1,935	44,576±12,815
2^nd^ challenge dose (per naris) (mean±SD)	13,830±4,504	n/a

### Nasal washing and determination of carriage

NW samples were collected before and after challenge and processed as previously described [30]. Briefly, a single aliquot of 5 ml normal saline was instilled into each naris with the subject seated leaning back supported at 45° to the horizontal. At instillation, the subject was invited to hold their breath and push gently on the back of the upper teeth with their tongue. Saline was held in the nasopharynx for 5 seconds and then poured out by gently tipping the head forward while holding a Petri dish under the nose. The samples collected from each naris were pooled. This process was performed once for the 6B cohort but 3 times for the 23F cohort. Samples were transferred immediately to the laboratory for processing. The first pooled aliquot obtained from each NW visit in both cohorts was used to determine carriage status (all negative except for one volunteer) and pneumococcal specific immunoglobulin analysis. NW samples were spun at 3345 g for 10 minutes and the supernatant stored at −80°C. The pellet was re-suspended in 100 µl of skim milk tryptone glucose glycerol (STGG) preservative medium prior to plating. 25 µl was plated onto Columbia Horse Blood Agar (Oxoid) with gentamicin (Sigma) and the remainder diluted to 1 ml with STGG and plated (50 µl) on blood and chocolate agar (Oxoid) for determination of co-colonising flora. Remaining STGG samples were stored at −80° for long term storage [31] and confirmation if needed. Plates were inspected after 24 hours incubation at 37°C, 5% CO_2_ and alpha haemolytic, draughtsman-like colonies were sub-cultured to determine pneumococcal phenotype. Optochin sensitivity, bile solubility and latex agglutination testing were performed to confirm pneumococcal phenotype. Subjects in whom pneumococci were not detected from NW samples collected on at least 2 consecutive visits were defined as non-colonised. Colonised subjects are defined as subjects in whom pneumococci was recovered from any nasal wash.

The numbers of lymphocytes in NW were too few for analysis of cognate antigen specific T cell responses using flow cytometry. We measured the NW total cell count and also examined stained cytospin slides for increased numbers of cellular effectors and mucus deposits. Total cell counts in NW were determined from pooled aliquots 2 and 3 from the 23F cohort. Cells were pelleted at 400 g and resuspended in PBS. Cell counts were performed using 10 µl of cell suspension and a haemocytometer, with the final count given per ml of NW fluid returned. The remainder was pelleted as above and re-suspended in 50 µl of PBS and centrifuged onto a microscope slide using a cytocentrifuge (ThermoFisher Scientific) for 5 mins at 450 rpm. For the 6B cohort a representative sample of 10 µl was taken from STGG preparations (described above). These samples were made up to 50 µl with PBS and cytospins were prepared as described. Microscope slides were allowed to air dry before staining with Hemacolor rapid staining set (Merck, Germany) according to the manufacturer's instructions. An arbitrary semi-quantitative scoring system was used to assign whether an increase, decrease or no change had occurred.

### Blood processing

Peripheral blood mononuclear cells (PBMCs) were isolated from volunteer samples using Lymphoprep (Axis Shield, UK) according to the manufacturer's instructions. PBMCs were incubated at 37°C, 5% CO_2_ at a concentration of 5×10^5^ cells per ml/well in a 48-well plate in RPMI 1640 media with 2 mM L-glutamine (both Sigma-Aldrich) and 10% human AB serum (Invitrogen, UK). Cells were stimulated on the same day as collection.

Serum was obtained from clotted whole blood collected into serum monovettes (Sarstedt, UK). Samples were centrifuged at 2560 g for 7 minutes. Serum was taken and stored at −80°C for immunoglobulin analysis.

### BAL processing

Bronchoscopy was performed using topical local anaesthesia with minimal sedation and BAL collected as described previously [32]. Briefly, four aliquots, totalling 200 mls, of warm 0.9% saline was instilled and gently retrieved from a sub-segmental bronchus of the right middle lobe by gentle hand suction and the BAL placed into sterile pots on ice. BAL was processed as previously described [33]. Briefly, whole BAL was filtered through muslin to remove mucus and 3 ml of unprocessed BAL was stored at −80°C. The whole sample was centrifuged at 470 g for 10 minutes. BAL supernatant was stored at −80°C for immunoglobulin analysis. BAL cells were re-suspended in RPMI 1640 media as described for PBMCs with the addition of antibiotics (penicillin 40 U/ml, streptomycin 40 µg/ml, neomycin 80 µg/ml (all P4083, Sigma-Aldrich) and 0.5 µg/ml of amphotericin B (A2942, Sigma-Aldrich). Total BAL macrophages and lymphocytes were counted and 5×10^4^ cells were centrifuged onto cytospin slides for differential staining as described. Remaining cells were then plated out into standard tissue culture plates to allow macrophages to adhere for 3 hours at 37°C, 5% CO_2_. Non-adherent cells were collected, washed and the pellet re-suspended in media as described for PBMCs. These BAL cells were then placed in 48-well plates at 37°C, 5% CO_2_ and stimulated on the same day as collection as described below for PBMC and BAL cells. For each volunteer we ensured that paired BAL cells collected at each bronchoscopy were processed and incubated using the same serum lot number and lymphocyte count/well during stimulation.

### Pneumococcal whole cell ELISA

Whole 23F (P833) and 6B (BHN418) strains were used as the capture antigen to determine the antibody titer to the pneumococcus in collected NW, BAL and serum. The whole cell ELISA assay was performed as previously described [34]. Briefly, bacteria stocks were grown in Todd Hewitt broth containing 0.5% yeast extract (THY) to an optical density (OD_600 nm_) of 0.5. The bacterial pellet collected after centrifugation was then re-suspended in carbonate-bicarbonate buffer to an OD_600 nm_ of 1. This bacterial suspension was added to 96-well Maxisorp plates (Nunc) (50 µl per well) and the plates were incubated overnight at room temperature. After washing with PBS containing 0.05% Tween 20 (PBS-T), wells were blocked with PBS containing 1% bovine serum albumin (BSA). Eight-fold serial dilutions of samples in 0.1% BSA were added and incubated for 2 hours at room temperature in triplicate. Antigen-specific IgG and IgA antibodies were detected using alkaline-phosphatase conjugated goat anti-human IgG (Sigma) and biotin conjugated goat anti-human IgA (AbD serotec, UK), respectively, followed by streptavidin-alkaline phosphatase (AbD serotec, UK). 0.5 mg/ml of p-nitrophenyl phosphate (PNPP) was added as a substrate. The OD was measured at 405 nm using a FLUOstar Omega (BMG Labtech, UK). The assigned titer value was determined as the last dilution in which OD is above 0.1.

### Measurement of anti-pneumococcal polysaccharide antibodies by ELISA

Anti-pneumococcal capsular polysaccharide antibodies were determined using the internationally standardised method and reagents [35]. Briefly, 96-well ELISA plates were coated using 5 µg/ml of purified polysaccharides 6B or 23F (Statens Serum Institute) for 5 hours at 37°C. Wells were blocked with 10% fetal bovine serum in PBS (PBS-F) for 1 hour at 37°C. Plates were washed 3 times with PBS-T between each step. Samples were diluted in PBS-F containing 10 µg/ml cell wall polysaccharide mixture (CWPS Multi, Statens Serum Institute) and incubated for 30 minutes at 37°C. When CWPS Multi is used, separate adsorption with the 22F capsule, is not required. 89-SF5 reference serum received from U.S. Food and Drug Administration was used as a standard. Diluted/adsorbed samples were then transferred to pre-coated plates and incubated overnight at 4°C. Bound antibodies were detected using alkaline phosphatase conjugated goat anti-human IgG (Sigma) for 2 hours at room temperature. 0.5 mg/ml of p-nitrophenyl phosphate (PNPP) was added as a substrate. Optical densities were measured at 405 nm using a FLUOstar Omega microplate reader (BMG Labtech, UK). All samples were run in triplicate in four dilutions. Results are expressed as µg/ml calculated using the assigned IgG concentrations in reference serum 89-SF5.

### Measurement of anti-pneumococcal protein antibodies

The recombinant proteins PspA (clade1) [36] and PspC (group 5) [37] (GenBank accession numbers AY082387 and EF424119.1) were kindly provided by Dr Eliane Miyaji (Butantan Institute, Brazil). Pneumolysin toxoid (PdB) was provided by Prof. Aras Kadioglu (University of Leicester, UK) and PsaA by Dr Eddie Ades (CDC Atlanta, GA). Purified proteins were used to coat ELISA plates at 1 µg/ml overnight. The assay was then performed as described above for the pneumococcal whole cell ELISA. Anti-PspA and anti-PspC concentrations were calculated using reference serum samples with known concentrations assigned in the laboratory of Prof. David Briles and Prof. Susan Hollingshead for PspA, (both University of Alabama, USA) and Prof. Helena Käyhty for PspC, (National Institute for Health and Welfare, Helsinki, Finland). All samples were run in triplicate in four dilutions. Anti-PdB and anti-PsaA concentrations are expressed in arbitrary units per ml calculated using a standard serum sample.

### Western blot analysis

We prepared whole cell extracts (WCE) and choline chloride extracts (CCE) from the inoculated 23F pneumococcal strain grown in THY to 0.6 OD_600 nm_. The cell pellet from 50 ml culture was lysed in a solution containing 0.01% sodium dodecyl sulfate, 0.1% sodium desoxycholate, 0.15 M sodium citrate [38] or 2% choline chloride [39], respectively. Choline chloride extraction is more effective at releasing the choline binding proteins on the pneumococcal surface. Protein extracts (5 µg of WCE and 3 µg of CCE) were separated in SDS-PAGE and transferred to a nitrocellulose membrane. Individual lanes were incubated at 25°C for 6 h with matched pre- and post-challenge NW samples (1∶25 diluted) from inoculated volunteers. Protein-specific antibodies were detected using goat anti-human IgG-HRP (Sigma-Aldrich). Detection was performed using an enhanced chemiluminescence (ECL) kit (GE Healthcare). Comparisons were only made between experiments in which the antibody incubation and development of membranes were performed at the same time to control for assay variation.

### Preparation of pneumococci for cell stimulation experiments

Serotype 23F and 6B *S. pneumoniae* were grown to mid-log phase in Vegitone broth (Oxoid) at 37°C, 5% CO_2_. To obtain pneumococcal culture supernatant, broth cultures were centrifuged and the supernatant was filtered first through 0.45 µm pore size and then 0.2 µm filters to remove remaining bacteria. Filtrate was then concentrated 10-fold by adding to pre-sterilised 10 K molecular weight cut-off Vivaspin concentrators (VWR) and centrifuging at 836 g for 25 mins at room temperature. This processed cell supernatant from culture broth defined here as ‘23F c/s’ or ‘6B c/s’ was stored at −20°C. Sterile Vegitone broth was processed under the same conditions and used as a control for stimulation experiments (‘vehicle’). Mid-log phase 6B pellet was killed by heat treatment for 30 minutes at 56°C and kept at −20°C in single use aliquots in PBS. The protein content of 23F c/s, 6B c/s, 6B pellet and vehicle was determined by Bradford assay. In addition, ethanol-killed acapsulate derived type 2 *S. pneumoniae* (gift of Prof. R Malley, Boston, USA) at a concentration of 1×10^6^ cells/ml was used as an *in vitro* pneumococcal challenge, relevant to the development of that product as a novel vaccine [10].

### PBMC and BAL cell stimulation

We collected PBMC and BAL samples from 19 volunteers before and after challenge with 23F or 6B to determine whether nasal challenge with pneumococcus was associated with alteration in pneumococcal antigen-specific memory CD4 T cell responses in either compartment. PBMC and BAL cells from volunteers challenged with 23F were stimulated *in vitro* with the following antigens: 23F c/s (0.395 µg/ml), vehicle (0.26 µg/ml), 10^6^/ml whole pneumococcal cells (acapsulate derived type 2 donated and prepared [33] by Prof. Malley), 0.45 µg/ml heat-inactivated influenza vaccine (Split Virion, Sanofi Pasteur 2009/2010 strains) or left untreated (‘NS’). All experiments were performed in a volume of 1 ml in 48-well plates. PBMC and BAL cells from volunteers challenged with 6B were similarly stimulated *in vitro* with the following antigens: heat killed 6B whole cells (1.0 µg/ml), 6B c/s (13 µg/ml of which 4.2 µg/ml was pneumococcal protein), vehicle (13 µg/ml), 10^6^/ml whole pneumococcal cells (acapsulate derived type 2 donated by Prof. Malley), 0.45 µg/ml heat-inactivated influenza (Split Virion, Sanofi Pasteur 2010/2011 strains) or left untreated (‘NS’). Staphylococcal enterotoxin B (Sigma-Aldrich) was used at 0.5 µg/ml (final concentration) as a positive control. Antigen titration experiments were performed to determine the optimum concentration required for stimulation. Cells were incubated for 2 hours at 37°C, 5% CO2. After 2 hours, 1 µl Brefeldin A (BD Biosciences, UK) was added and incubated for a further 16 hours before harvesting and staining for the presence of intracellular cytokines by flow cytometry.

### Intracellular cytokine staining and flow cytometry analysis

Cells challenged with antigen as above were spun to a pellet, supernatant discarded and the cells stained for flow cytometry. Cells were stained with Vivid according to the manufacturer's instructions (Invitrogen) to allow discrimination between viable and non-viable cells. Cells were then stained for CD3, CD4 and CD45RO using the following mouse anti-human monoclonal antibodies, APC conjugated CD3, APC-H7 conjugated CD4, PE-Cy7 conjugated CD45RO (all Becton Dickinson) on ice. Cells were fixed and permeabilised (Cytofix/Perm) according to the manufacturer's instructions (BD Biosciences) then stained for intracellular IFNγ, TNF and IL-17 using mouse anti-human antibodies: AF_700_ conjugated IFN-γ, AF_488_ conjugated TNF, PE conjugated IL-17 (all BD Biosciences, UK). Cells were fixed prior to acquisition on a BD LSR2 flow cytometer (Becton Dickinson, UK). We gated viable, memory CD4 T cells (CD4+CD45RO+) and performed a Boolean gating strategy (data not shown) to identify the proportion of TNF, IFNγ and IL-17 producing cells (or combinations thereof) following *in vitro* stimulation with Influenza (positive control), whole pneumococci or pneumococcal culture supernatant from the respective challenge strain. In order to correct for inter-subject variation in the response to the Vegitone broth, all culture supernatant stimulated data were corrected by subtracting the percentage of CD4+CD45RO+ cells producing cytokine when cultured with Vegitone media alone (“vehicle”) from the pneumococcal antigen stimulated response. Responses to media alone were subtracted from Influenza or whole cell stimulated cells to determine specific responses.

### Statistical analysis

Immunoglobulin and flow cytometry data were tested to determine the distribution of the data. Data with a normal distribution (Shapiro Wilks) were compared with parametric tests (paired students' t-test for before vs after inoculation) and for data not following a normal distribution; the Wilcoxon-matched pairs test was used. Flow cytometry data were analysed using FlowJo software version 7.6 (Treestar Oregon, USA). Graph and statistical analysis was performed using GraphPad prism version 5.0 (California, USA). Differences were considered significant if p≤0.05.

## Results

### Recruitment and human pneumococcal challenge

Twenty volunteers were recruited and inoculated per protocol as shown in Figure 1 with no adverse effects. One subject in the 23F cohort established experimental colonisation as a result of the inoculation and is excluded from further description in this study. In the first cohort 8 subjects received two doses of 23F mean dose 8,791 cfu/naris (SD±1,935 cfu/naris) and 13,830 cfu/naris (SD±4,504 cfu/naris), given 2 weeks apart (Figure 1 A and Table 1). In our second cohort we challenged 11 volunteers with a single, mean dose of 44,576 6B cfu/naris (SD±12,815 cfu/naris) (Figure 1 B and Table 1). These remaining nineteen study volunteers did not establish colonisation following pneumococcal challenge, contrary to our initial expectations. We did not detect pneumococci in any nasal washes obtained from these nineteen volunteers. Details of the study participants who were challenged with pneumococci are given in Table 1 for both 23F and 6B cohorts.

Nasal wash volumes recovered were a mean of 5.9 ml (range 2.3 to 8.5 ml) for the 23F cohort and 6.5 ml for the 6B cohort (range 4 to 8.1 ml). BAL volumes with cell counts are shown in Table S1.

### Intra-nasal pneumococcal challenge increases mucosal pneumococcal specific IgG and IgA

**Figure 2:**
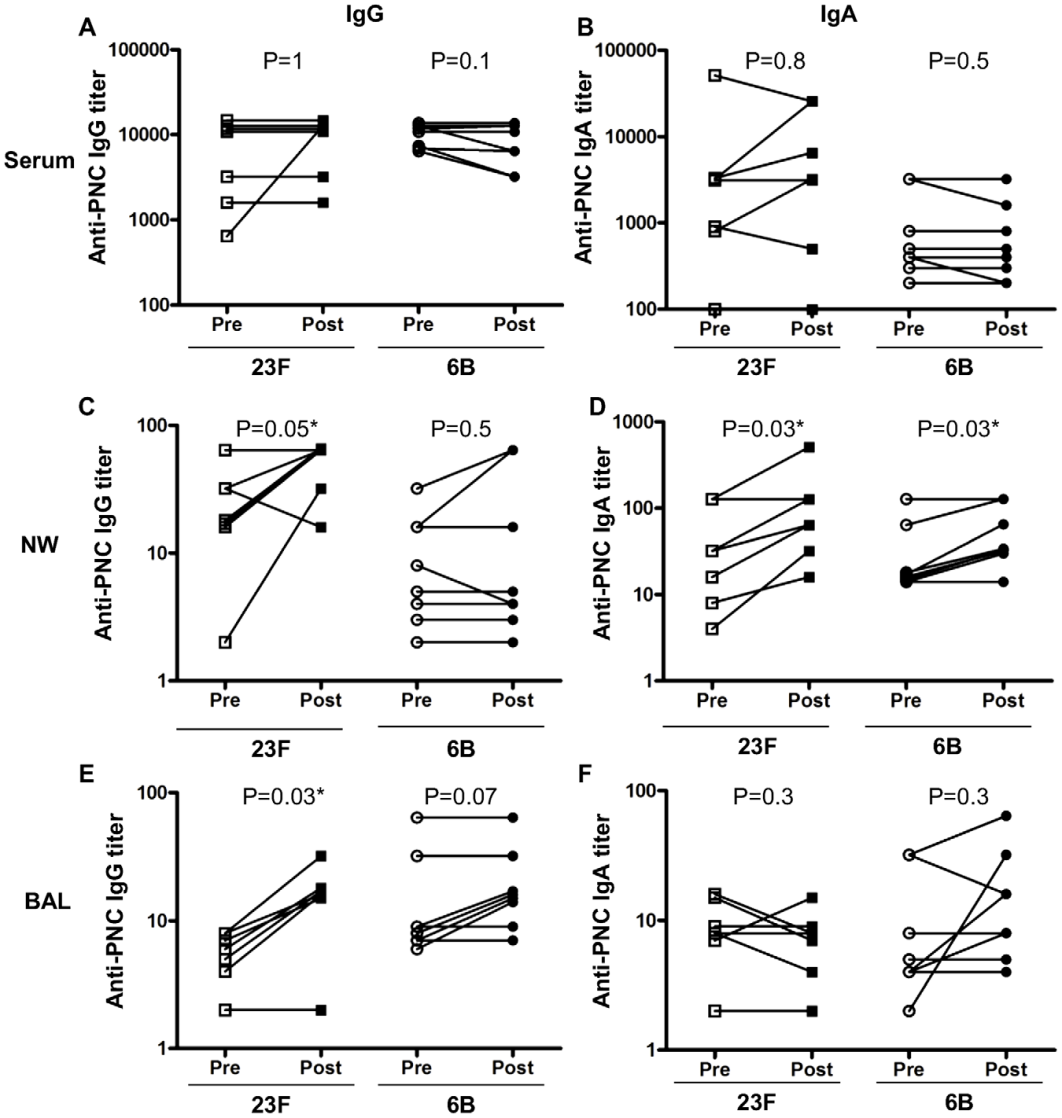
IgG and IgA responses to whole cell 23F or 6B pneumococci following 23F or 6B challenge, respectively. ELISAs were performed using 23F or 6B pneumococci as targets to measure specific IgG (A, C and E) and IgA (B, D and F) titers in serum (A and B), NW (C and D) and BAL (E and F). Values shown are the mean antibody titers (of triplicates), pre and post 23F (*n* = 7) or 6B (*n* = 8) challenge (x-axis). *represents statistical significance between pre- and post-inoculation antibody titers.

Pneumococcal whole cell ELISA data were compared before and after pneumococcal challenge in 3 compartments (NW, BAL and serum) using samples from the first 7 volunteers for the 23F cohort and 8 volunteers in the 6B cohort as shown in Figure 2. Three volunteers were excluded from the 6B cohort as a full set of paired samples were not obtained. In each cohort, the relevant challenge pneumococcal strain was used as the ELISA target antigen. Whole cell ELISA using 23F and 6B pneumococcus detected antigen-specific IgG and IgA in all NW, BAL and serum samples both before and after inoculating challenge. Antigen-specific titers of pneumococcal specific IgG were much higher in serum than in NW (200-fold) or BAL (1000-fold). Similarly, serum levels of pneumococcal specific IgA were much higher than in NW (10-fold for 6B and 100-fold for 23F) and BAL (100-fold for 6B and 1000-fold for 23F). There was no association between volume of fluid returned and immunoglobulin titer for either NW or BAL samples.

Comparisons before and after nasal inoculation with bacteria showed that there was no change in pneumococcus-specific IgG or IgA in serum following inoculation with either 23F or 6B as shown in Figures 2A and 2B. There was a significant increase in both IgG (pre 25.4±7.5 vs post 52.5±7.5, p = 0.05) and IgA titers (pre 47.5±17.9 vs post 122±57.6, p = 0.03) in NW following 23F inoculation, and a significant increase in IgA in NW following 6B inoculation (pre 36±14.4 vs post 58±15.9, p = 0.03) as shown in Figures 2C and 2D. BAL data showed a significant increase in anti-pneumococcal IgG titer following 23F (pre 6±2.5 vs post 16.2±8.6, p = 0.03) but not 6B challenge (Figure 2E). Anti-pneumococcal IgA responses in BAL before and after 23F or 6B challenge were unaltered (Figure 2F). In summary, intranasal inoculation of bacteria was associated with increased NW (IgG and IgA) and BAL (IgG only) immunoglobulin responses but no change in serum levels. These observations were dependent on whether the challenge was with 23F (NW and BAL) or 6B (NW IgA only) pneumococcal serotype.

### Increased pneumococcal specific IgG and IgA responses are directed towards pneumococcal proteins and not capsular polysaccharide

We next determined which pneumococcal antigenic targets were responsible for the increased antibody responses. We measured anti-23F or 6B capsular polysaccharide responses before and after challenge with the respective challenge strain in NW, BAL and serum. There were no significant differences in 23F or 6B anti-capsular polysaccharide responses before and after challenge in either cohort (Figure S1 A–C).

**Figure 3:**
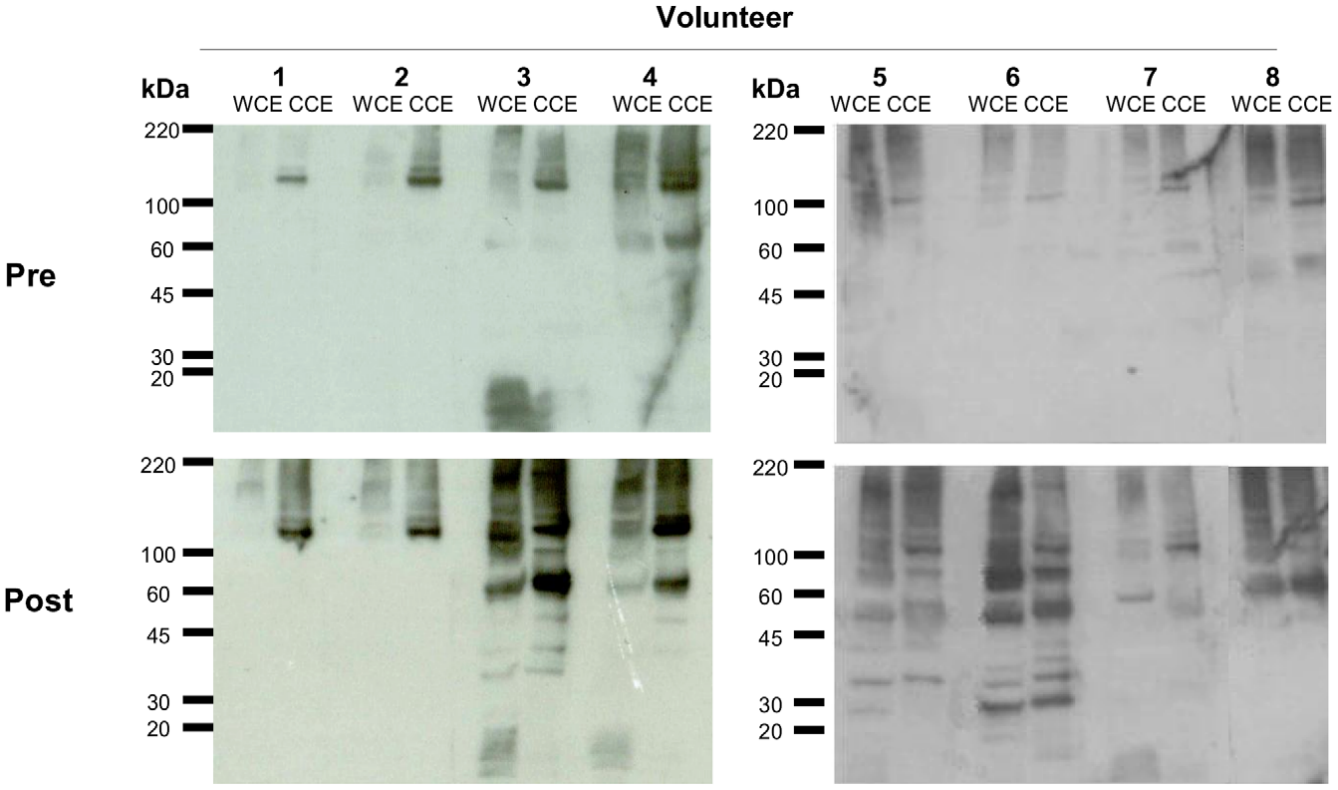
Nasal wash IgG binding to pneumococcal proteins before and after 23F pneumococcal challenge by Western blot. Pre- and post- 23F challenge NW samples from 8 volunteers were used to detect pneumococcal proteins present in whole cell (WCE) and choline chloride extracts (CCE) of the challenge strain.

We followed up our observations in Figure 2, showing a significant difference in IgG titer before and after 23F but not 6B challenge, by performing Western blots to compare IgG responses to 23F pneumococcal proteins on the same NW sample set. Figure 3 shows results from NW taken from 8 subjects before (Figure 3 Pre) and after (Figure 3 Post) nasal challenge with 23F. There was more antigenic protein in each gel following CCE compared to WCE. The dominant protein antigen seen in all NW samples before challenge was a CCE band migrating to 110–150 kDa. Following challenge with 23F (Figure 3 Post) there was a general increase in the overall level of NW antibody binding in both WCE and CCE lanes in all NW samples. There was particularly increased reactivity towards the 110–150 kDa band in all but one subjects and a new band slightly above 60 kDa in 5/8 donors (Figure 3 Post). These data indicate an increase in IgG response towards pneumococcal proteins following 23F exposure.

### Pneumococcal protein specific IgG is increased in NW following 6B but not 23F challenge

**Figure 4:**
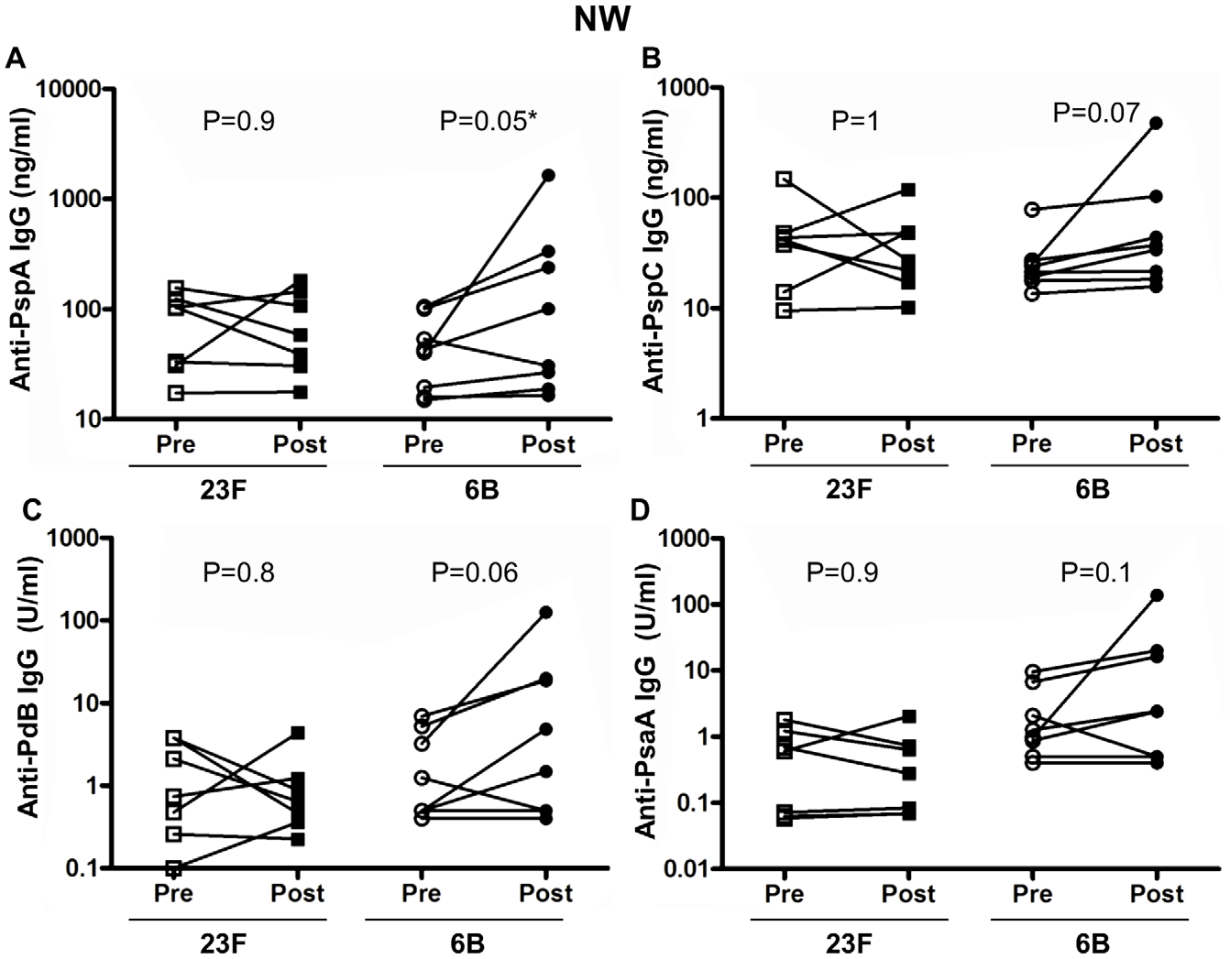
Nasal wash IgG response to pneumococcal purified protein antigens. ELISAs were performed using pneumococcal antigens PspA (A), PspC (B), PdB (C) and PsaA (D) to determine specific IgG in subject's pre and post 23F (*n* = 7) or 6B (*n* = 8) pneumococcal challenge (x-axis). Values shown are the mean antibody concentration (of triplicates). Antibodies to PspA and PspC are expressed in µg/ml and antibodies to PdB and PsaA are expressed in arbitrary units/ml (y-axis). *represents statistical significance between pre- and post-challenge antibody levels.

We next used purified proteins in an ELISA to quantitatively determine the specific pneumococcal proteins which elicited increased total pneumococcal specific IgG responses in NW following bacterial challenge. We measured anti-PspA, PspC, PdB and PsaA IgG (Figure 4) levels in both 23F and 6B pneumococcal challenge cohorts before and after challenge. In the 23F challenge cohort we did not detect any difference in the concentration of anti-PspA (Figure 4A), PspC (Figure 4B), PdB (Figure 4C) or PsaA (Figure 4D) IgG before and after challenge. In the 6B challenge cohort, we detected a significant rise in the mean concentration of anti-PspA (pre 49.2±12.7 vs post 301.5±196.5, p = 0.05) (Figure 4A). The mean concentration of anti-PspC (pre 28.2±7.3 vs post 93.8±55.5, p = 0.07) (Figure 4B), anti-PdB (pre 2.3±0.8 vs post 21.4±15.12, p = 0.06) (Figure 4C) and anti-PsaA (Figure 4D) (pre 2.8±1.2 vs post 22.5±16.6, p = 0.1) antibody were all higher post 6B challenge compared to pre-challenge samples but these differences were not statistically significant. The Western blot and these ELISA data suggest that the antibody response to whole pneumococci extends beyond these 4 studied antigenic proteins.

### Intra-nasal 23F pneumococcal challenge increases anti-PspA IgG in BAL

**Figure 5:**
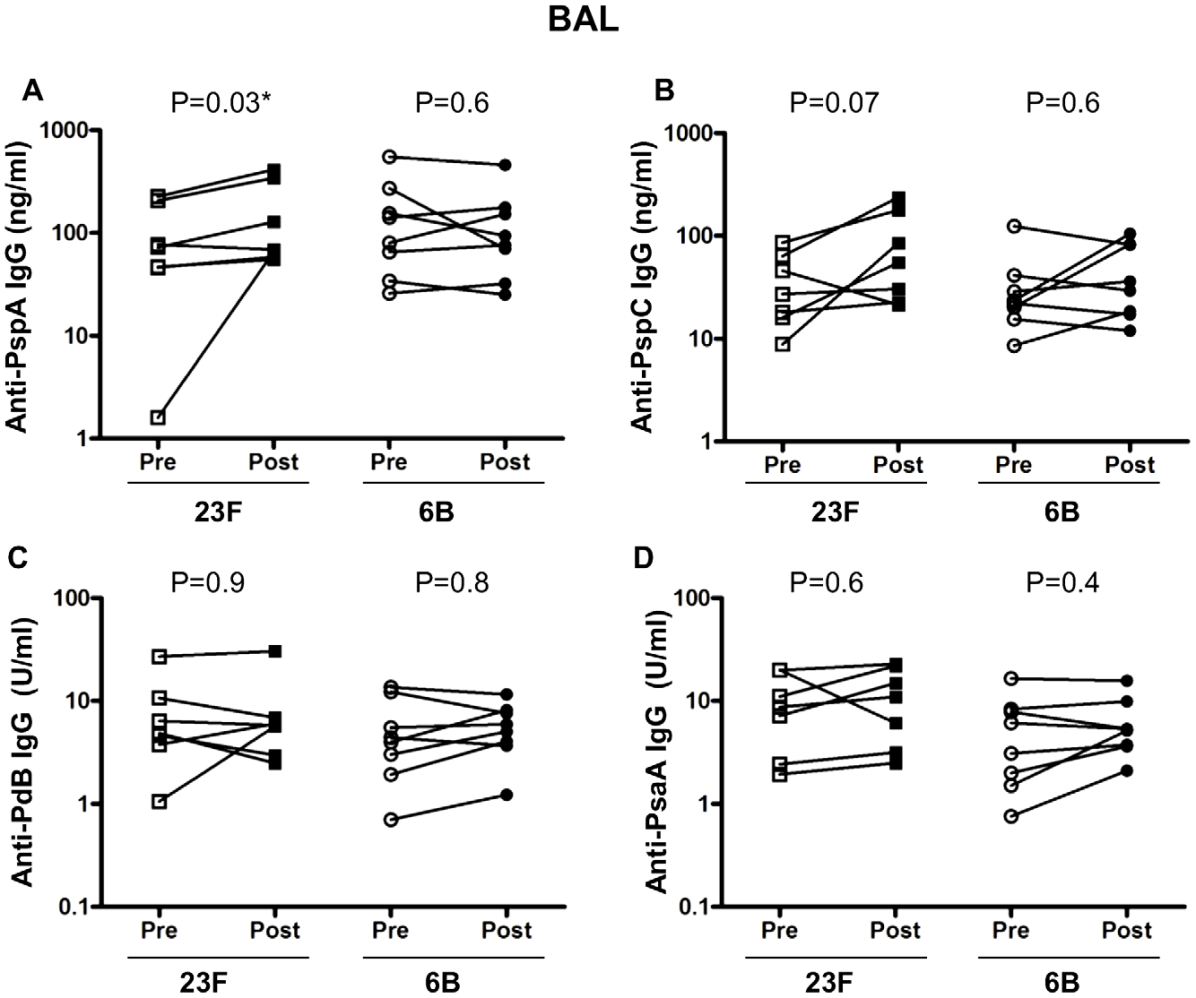
Bronchoalveolar lavage IgG response to pneumococcal antigens. ELISAs were performed using pneumococcal antigens PspA (A), PspC (B), PdB (C) and PsaA (D) to determine specific IgG in subjects pre and post 23F (*n* = 7) or 6B (*n* = 8) pneumococcal challenge (x-axis). Values shown are the mean antibody concentration (of triplicates). Antibodies to PspA and PspC are expressed in µg/ml and antibodies to PdB and PsaA are expressed in arbitrary units/ml (y-axis). *represents statistical significance between pre- and post-challenge antibody levels.

Bacterial challenge with type 23F pneumococcus was associated with increased BAL IgG titer in pneumococcal whole cell ELISA but not with increased anti-capsular BAL IgG. We therefore measured anti-pneumococcal protein IgG levels in BAL samples following 23F challenge. We measured anti-PspA, PspC, PdB and PsaA IgG responses in BAL (Figure 5) for both challenge cohorts. There was a significant increase in the concentration of anti-PspA specific IgG in BAL following challenge with 23F (pre 96.4±32.2 vs post 161.7±57, p = 0.03) but not 6B (Figure 5A). A higher concentration of anti-PspC IgG in BAL following challenge with 23F (pre 37.9±10.7 vs post 90.2±32.3, p = 0.07) was observed (Figure 5B) but was not statistically significant. There were no differences in the concentration of anti-PdB (Figure 5C) and PsaA (Figure 5D) IgG in BAL before and after challenge with 23F. There were no differences in protein specific responses in BAL before and after 6B challenge (Figure 5A–D).

### Pneumococcal 23F challenge decreases serum anti-PspA

**Figure 6:**
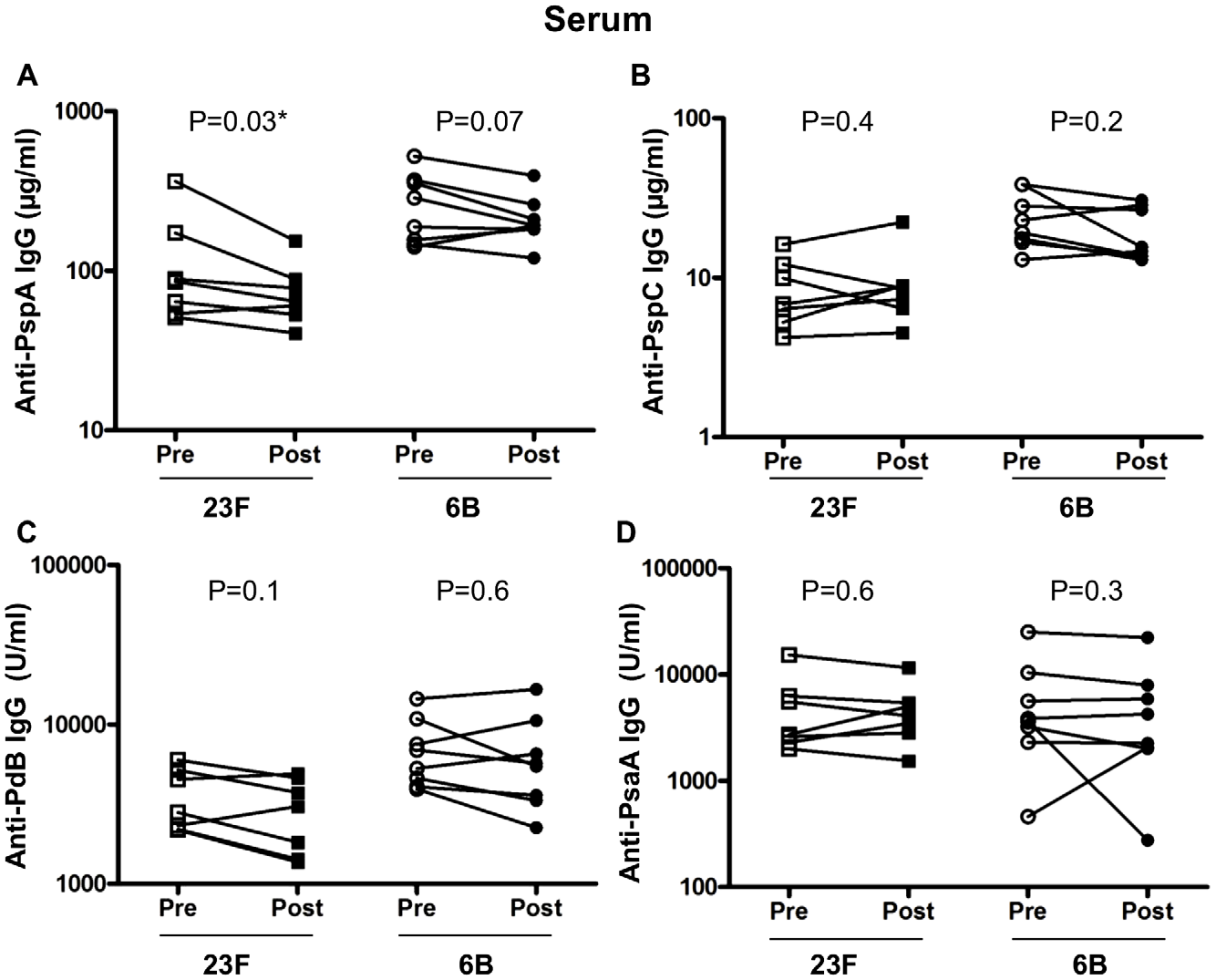
Serum IgG response to pneumococcal antigens. ELISAs were performed using pneumococcal antigens PspA (A), PspC (B), PdB (C) and PsaA (D) to determine specific IgG in subjects pre and post 23F (*n* = 7) or 6B (*n* = 8) pneumococcal challenge (x-axis). Values shown are the mean antibody concentration (of triplicates). Antibodies to PspA and PspC are expressed in µg/ml and antibodies to PdB and PsaA are expressed in arbitrary units/ml (y-axis). *represents statistical significance between pre- and post-challenge antibody levels.

Although there were no changes in serum observed following bacterial challenge using either whole cell ELISA or capsular polysaccharide ELISA, we measured anti-PspA, PspC, PdB and PsaA IgG in serum before and after challenge with 23F or 6B (Figure 6). In contrast to the increased anti-PspA measured in BAL following 23F challenge there was a significant *decrease* in the concentration of serum anti-PspA following challenge with 23F (serum pre 125.6±42 vs serum post 76.9±14, p = 0.03) but not 6B (Figure 6A). The concentration of anti-PspC (Figure 6B), PdB (Figure 6C) and PsaA (Figure 6D) were similar before and after challenge for both 23F and 6B cohorts.

### Pneumococcal challenge shows evidence of mild inflammation

We next compared the cellularity and cellular responses in NW samples obtained from subjects before and after pneumococcal challenge from both 23F (first dose only) and 6B cohorts.

NW sample cellularity was low and variable (range 0–9300 total cells) in samples collected. There was no difference in total cell yield before and after challenge for the 23F cohort (Figure S2). Increased levels of cellular effectors (neutrophils and mononuclear cells) and/or granular mucus deposits (evidence of inflammation) compared to baseline were seen on cytospins of NW preparations in 4/8 volunteers 2 days after 23F challenge and in 2/8 volunteers at 4 days. IL-17 ELISA (eBioscience, UK, Catalogue 88-7176-22) performed according to the manufacturer's instructions on NW samples from 4 volunteers showed no detectable cytokine before and after 23F challenge. In the 6B cohort 5/7 samples showed increased cellular effectors and/or granular mucus deposits in samples taken immediately after 6B challenge compared with before challenge.

### Pneumococcal challenge is associated with altered mucosal T cell memory in BAL but no increase in antigen-specific T cell responses before and after challenge

There were no differences in BAL volume and total cell yield before and after 23F or 6B challenge (Table S1). In the cohort challenged with 23F, there was a significantly higher total lymphocyte count (mean±SD Pre 1.9±0.75×10^6^ vs post 3.2±1.2×10^6^ p = 0.006) after challenge compared to before in BAL. The percentage of total T cells that were of the memory CD4 phenotype was also significantly higher after challenge compared to before (mean%±SD, Pre 40.05±20.6 vs post 48.47±14.7, p = 0.048). Total BAL lymphocyte counts showed correlation with the BAL anti-23F whole cell antibody titre (p = 0.01, Pearson correlation) and anti-PspA concentration (p = 0.03, Pearson correlation) for the 23F but not the 6B cohort (Figure S3). In the cohort challenged with 6B, there was no increase in total lymphocyte count, no increase in % memory CD4 T cells and no association of lymphocyte numbers with anti-pneumococcal IgG by whole cell ELISA (Figure S3).

**Figure 7:**
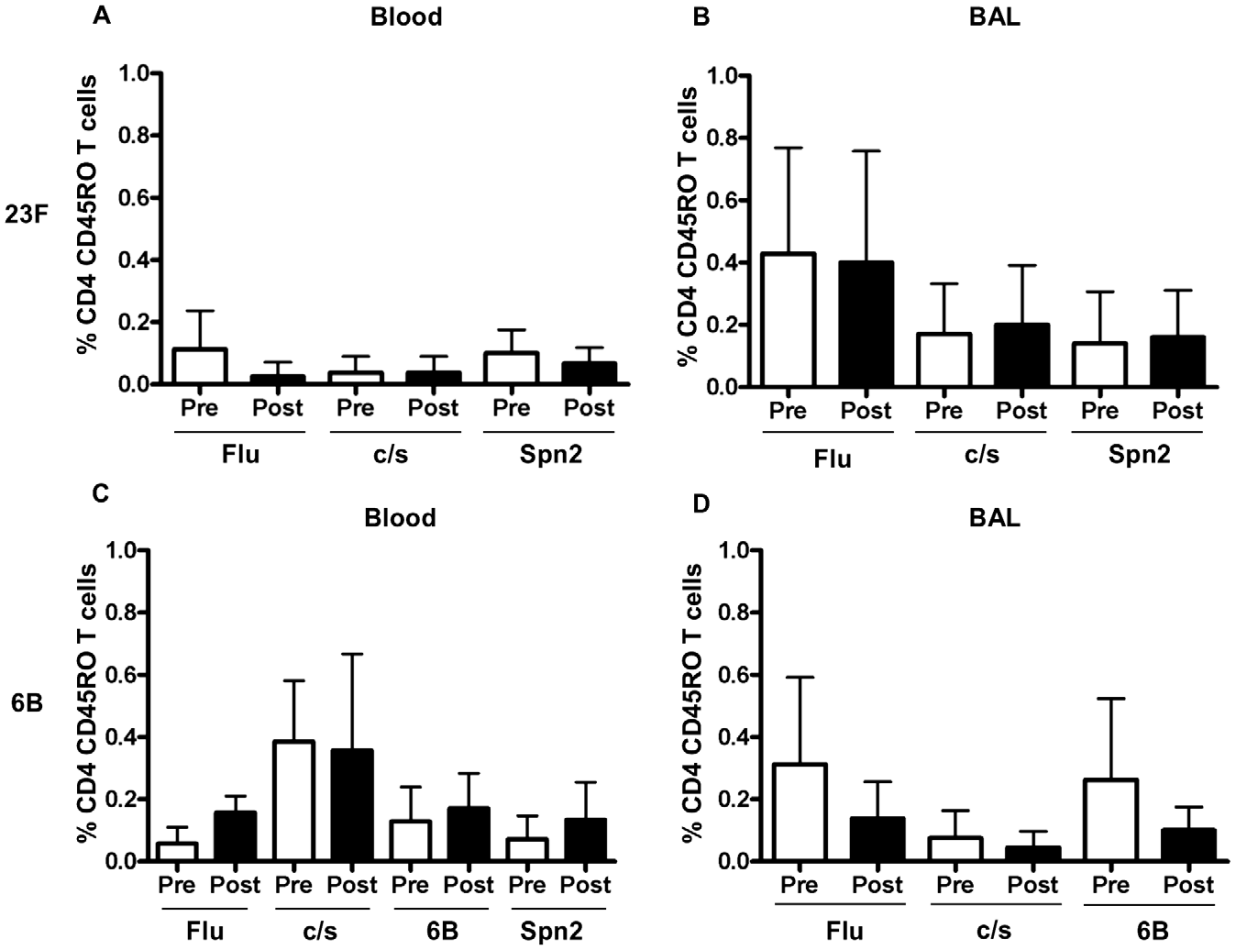
Frequency of antigen specific CD4 T cell responses before and after 23F or 6B challenge. Blood (A and C) and BAL (B and D) responses are shown pre and post 23F (A and B) or 6B (C and D) challenge. The proportion of flu and pneumococcal specific (x-axis) CD4+CD45RO+ T cells producing either TNF ± IFNγ ± IL-17 (or combinations thereof) (y-axis) were analysed by multi-parameter flow cytometry. Shown are mean values ± SD of at least 6 paired samples.

The data for antigen-specific T cell responses (baseline corrected) are shown in Figure 7 as the percentage of memory CD4 T cells producing at least one cytokine (of IFN-γ, TNF or IL-17) before and after the subject was intra-nasally inoculated with pneumococci.

Antigen-specific responses, to influenza and pneumococcal preparations, compared to non-stimulated cells, were seen to a varying degree in blood and BAL from both cohorts *before* challenge. In the 23F cohort, significant antigen-specific responses (compared with non-stimulated control) were seen to influenza in BAL (mean%±SD, 0.85±0.66 vs non-stimulated 0.42±0.36 p = 0.03, *n* = 7) and blood (0.15±0.05 vs control 0.05±0.07, p = 0.03, *n* = 8). There were no significant responses to pneumococcal antigens before inoculation in blood or BAL but there were significant responses to 23 c/s *after* inoculation in BAL (0.55±0.28 vs vehicle 0.35±0.16, p = 0.032, *n* = 8).

In the 6B cohort, significant antigen-specific responses (compared to non-stimulated control) were seen to influenza in BAL (0.5±0.29 vs non-stimulated 0.19±0.09, p = 0.02) but not blood. We also detected significant responses to 6B whole pneumococci but not 6B culture supernatant in BAL (0.40±0.26 vs control 0.18±0.09 p = 0.05) before pneumococcal challenge. In blood we detected significant responses to 6B c/s (0.62±0.26 vs vehicle 0.24±0.09, p = 0.0020) but not 6B whole pneumococci before challenge.

Paired comparisons, before and after challenge, did not show any significant differences in the proportion of pneumococcal specific cytokine producing memory CD4 T cells before and after challenge with 23F (Figure 7A blood and 7B BAL) or 6B (Figure 7C blood and 7D BAL) in blood or BAL.

## Discussion

We anticipated that a low dose of live pneumococci delivered intra-nasally would lead to colonisation and thus augmented mucosal and systemic responses to pneumococci. Colonisation was not achieved in this study but we have shown that human inoculation with a low dose of live pneumococci elicits specific mucosal responses in the absence of carriage. Healthy adult volunteers with detectable humoral and cellular immunity to pneumococci prior to inoculation showed increased immunoglobulin responses in NW and BAL but not blood 6 weeks after experimental bacterial inoculation. In particular, concentrations of IgG measured following inoculation with 23F were increased compared to baseline observations in NW and BAL but not in serum using a whole bacteria ELISA. Concentrations of IgA measured following inoculation with 23F or 6B were increased compared to baseline observations in NW but not in BAL or serum. These responses did not include IgG or IgA to pneumococcal capsule. We measured IgG responses in NW, BAL and serum towards four pneumococcal proteins. We found that IgG anti-PspA was significantly elevated in NW (following 6B challenge) and BAL (following 23F challenge) and accounted in part to the elevated responses seen towards whole pneumococci. Inoculation was associated with an increase in the absolute count and percentage of mucosal memory CD4 T cells for the 23F cohort but not with an increase in paired antigen-specific cellular responses in either group.

Nasopharyngeal carriage of pneumococci occurs early in life, frequently in infancy [5] and less commonly in adult life [40]. The most dramatic reduction in carriage and also disease rate occurs in the second year of life [41] independent of capsular serotype [7]. Humoral and cellular responses to pneumococcal capsular polysaccharide and protein antigens develop during this time. These immunological developments contribute towards a much lower incidence of pneumococcal mucosal and invasive disease in older children and young adults than in infants [41], [42]. Repeated episodes of carriage and possibly pneumococcal exposure [13] are thought to boost these important defences.

Experimental human pneumococcal carriage (EHPC) in adults offers the platform to measure these responses [43]. EHPC in immune adults has been previously reported [25], [44] but there has been no previous quantitative report of the mucosal humoral and cellular immune response to challenge not resulting in carriage. Most adult exposures to pneumococcus do not result in carriage and indeed in our experimental pneumococcal inoculations, only one healthy adult progressed to carriage. This result is surprising given that the inoculated dose per naris for both cohorts in our study (Table 1) is similar (23F) or higher (6B) than the CFU dose described by McCool and Weiser [25] for their colonised (12,000±5,477/naris) and uncolonised (7,250±4,062/naris) groups. Colonisation success rate will depend on challenge dose but also on the sensitivity of detection, exposure to other non-/infectious agents, presence of co-morbidities, genetic factors, opacity (i.e. capsule expression) of the challenge strain [45] and study design.

The usefulness of the EHPC model depends on the sensitivity of assays for detection of carriage. Sample collection by nasal swab followed by enrichment for pneumococci using STGG medium and subsequent bacterial culture is the current gold standard method [46]. A previous EHPC model used throat and nasal swab or wash with standard bacteriological culture media [25]. We demonstrated that a NW sample is not only more comfortable for the volunteer but better than swabs for detecting upper respiratory flora [30] including pneumococcus [47]. We employed a method combining NW collection and direct plating followed by storage in STGG broth at −80°C [30]. A molecular approach to pneumococcal identification may yield greater sensitivity compared to culture [48], [49], but it does not distinguish between carriage, which implies the presence of viable pneumococci, and retention of bacterial antigen from non-viable cells. A sophisticated molecular approach [48] will resolve these issues in the future but we cannot rule out that some volunteers described here were colonised below the detection limit of the current gold standard method. The major strength of this study, however, is that it focused on the immunological impact of a potentially infecting dose of pneumococcus [25] on healthy adults with proven previous natural pneumococcal exposure. We have been able to describe responses in the inoculated site (nasopharynx), distal airway and circulating blood.

The onset of carriage and acquisition of immunity in infants has been successfully modelled using mice. These models have shown that intra-nasal inoculation using live [14], [16], [17], [50] or ethanol killed [10] pneumococci or recombinant proteins [37], [51]–[54] (some with adjuvant) can elicit humoral and cellular immune responses that protect against subsequent colonisation [14], [16]–[18] and/or disease [16], [17]. Murine models can dissect the relative contribution of humoral and cellular defence [55] but are a better model of infancy (naive response) than of adults with pre-existing memory responses. We have shown for the first time that levels of anti-protein IgG and IgA against pneumococus were boosted in both NW and BAL following intranasal challenge with pneumococci, though this depends on the serotype used. The implication of this finding is that exposure to low doses of pneumococcus is potentially immunising at the mucosal surface. A limitation of this study, however, is that the samples collected from the nasopharynx and lung are so dilute that functional measurement of the opsonophagocytic effect of the observed increase in immunoglobulin concentration is not currently possible. Previous studies in several laboratories including ours [56] have attempted to concentrate BAL in order to measure opsonic function. Concentration in this way allows binding to be determined but enhanced phagocytosis following immunisation has not been demonstrated.

It is interesting that there was no significant change in IgG or IgA to polysaccharide capsule following inoculation. This somewhat counter-intuitive observation is consistent with previous data showing a lack of BAL response to inhaled polysaccharide [57]. Human experimental colonisation however enhanced serum polysaccharide responses in 5/6 volunteers [25] suggesting colonisation has a greater systemic immunising effect compared to challenge. Polysaccharide responses are initiated in the spleen and plasma cells are found in very low numbers in BAL. In the current study we know that bacterial clearance from the nasopharynx was achieved in less than 2 days (probably more rapidly) and it is therefore reasonable to suggest that polysaccharide and bacterial protein were not presented to critical sites or cells to enhance systemic immunity. IgG to polysaccharide capsule is a critical defence mechanism against invasive pneumococcal disease but is less important in defence against carriage [18]. Our data suggest that experimental pneumococcal exposure without carriage does not augment this limb of systemic defence.

We showed altered mucosal responses to protein antigens, particularly choline bound proteins such as PspA. Using Western blot and whole cell ELISA, more IgG to pneumococcal protein was found after inoculation than before in both NW and BAL in the 23F cohort. The Western blots show a greater effect of inoculation on the choline binding proteins than on proteins extracted without choline chloride. Specific anti-protein ELISA was only able to detect a statistically significant difference in one of the 4 proteins tested (PspA) although NW IgG levels to the other three were all increased in the 6B challenged cohort. There are many pneumococcal surface-proteins that function as virulence factors and are sufficiently surface exposed to have vaccine potential. Some of these pneumococcal surface proteins, including PspA, PspC and PsaA, have been shown to have roles in adhesion and carriage and may vary in expression between pneumococcal isolates. Human anti-PspA responses elicited by intra-muscular vaccination with rPspA are protective against subsequent lethal challenge with heterologous pneumococci when passively transferred in murine models [12] and therefore the response seen in our 6B (NW) and 23F (BAL) cohort may be of functional significance. Both strains used in our study express PspA clade 1 similar to the PspA used for the ELISA. The absence of a NW response in the 23F cohort could be due to low PspA expression by the inoculated strain or adsorption of nasal antibodies to the inoculated bacteria. The concentration of IgG to PspA in mucosal samples is so diluted that direct functional assays or passive transfer experiments are unlikely to succeed. Alternatively, increased protection due to immunoglobulin responses in NW could be tested by repeated pneumococcal challenge of subjects with heterologous or homologous serotypes who develop experimental carriage and/or enhanced immunoglobulin responses on the first inoculation. This study is planned in our laboratory.

Murine models have demonstrated that pneumococcal colonisation or vaccination results in the generation of memory CD4 T and B cells in both the circulation and at the mucosa. The effector CD4 T cell phenotype is mixed but includes IL-17 [14], [37], [58] and/or IFNγ [37], [51] secreting T cells (T_h-17_/T_h-1_). NW samples from healthy adults had very low cell counts in this study which is not surprising given that mucosal immune cells are found in specialised sub-mucosal lymphoid aggregates, best investigated in humans by using surgical explants [59]. We were, however, able to demonstrate antigen-specific T cells producing cytokine in response to specific stimuli in BAL both before and after nasal inoculation. Pneumococcal challenge, however, did not alter the frequency of pneumococcal specific, cytokine secreting, CD4 T cells in BAL. The dose of pneumococci delivered to the lung in our non-colonised subjects is likely to have been very small and therefore the antigenic stimulus to lung during the inoculation period in our subjects will have been minimal. The lymphatic drainage of the nasopharynx is to the cervical lymph nodes whilst the lungs drain to the hilar nodes; we would therefore expect that antigen challenge of the nasopharynx would have limited impact on BAL lymphocytes. A recent murine model, however, has shown that viral upper respiratory tract infections lead to the migration of clonally-related populations of activated T cells to both upper *and* lower respiratory mucosal sites [60]. These data suggests that measuring the proportion and phenotype of antigen specific T cells in the BAL mucosal compartment in humans may be similar to the proportion and phenotype of effector T cell populations present in the sub-mucosa of the nasopharynx. In this study, we observed an increased absolute count of BAL lymphocytes and increased percentage of CD4 memory cells in BAL at second examination in the 23F cohort. This may reflect a response of the respiratory tract to antigen challenge as it only occurred in the 23F cohort and this cohort also had significant immunoglobulin anti-pneumococcal protein responses following challenge. It is also possible that the altered CD4 profile seen in 5/6 subjects in that cohort resulted from the non-specific activation caused by a previous BAL but this effect was not seen in the 6B cohort. Alternatively, the proportion of memory CD4 T cells in the 23F cohort (pre challenge) is lower than that in the 6B cohort (pre challenge) and therefore the increased recruitment and proportion of memory CD4 T cells may reflect a return to homeostatic levels with concomitant recruitment of antibody secreting plasma cells. There were no increases in antigen-specific CD4 T cell functional assays to suggest specific pneumococcal activation. Current studies in our laboratory aim to characterise the BAL lymphocyte response to experimental human pneumococcal carriage which will provide a more sustained antigen challenge to the lung.

We used two different pneumococcal strains and two different protocols in the experimental inoculations of two consecutive cohorts of volunteers. The results in each cohort were similar in that capsular responses were not detected in any compartment and enhanced immunoglobulin responses were not observed in blood. The study design was modified between 23F and 6B cohorts in an attempt to obtain colonisation and is different from that published previously by McCool and Weiser [25] likely influencing the results observed. In our study whole cell ELISA data showed significant changes only in the 23F cohort and changes in anti-PspA response were only seen in the 23F cohort when comparing before and after inoculation. This is most likely due to the double dose challenge procedure employed in the 23F cohort compared to the 6B cohort. Two doses are employed to first prime the immune system to vaccine and then second to boost specific responses. This has been performed successfully using rPspA (with alum as adjuvant) in humans leading to 100 fold increases of serum anti-PspA IgG in volunteers immunized with 125 µg of rPspA [12]. The rPspA given in this study, however, was intra-muscular and a larger dose. Mucosal sites were not examined for cellular or humoral responses. Differences between the 23F and 6B cohort described here in NW, BAL and serum cannot be ascribed to other confounding variables such as the after effects of BAL collection since this was obtained from both groups and non-specific activation would have to be maintained for at least 10 weeks before being detected in post samples. BAL collection before nasal exposure is a limitation of this study and is an important difference between our 23F challenge model and the model described by McCool and Weiser [25]. BAL collection may cause systemic [61] and local [62] side effects but these are usually short term (1–2 days) rather than long term (weeks) effects. We attempted to minimise the pro-inflammatory effect of BAL collection by maintaining a 2 week gap between sample collection and challenge. We cannot rule out the possibility that BAL collection approximately 2 weeks before challenge may have impaired our ability to obtain colonisation following challenge with 23F and 6B compared to the success rate of McCool and Weiser [25].

The different results obtained from our 23F and 6B cohorts may also be due to different antigenicity of the PspA found in the two inoculated strains; although both strains used for inoculation express similar PspA as that used for ELISA (all clade 1). We are now manufacturing the PspA from each strain in order to improve detection sensitivity but the implication of this pattern of findings is that immunogenicity varies between natural exposures independent of capsular type. In addition, the concentration of specific antibody required for mucosal protection may differ between natural and experimental exposure.

These data suggest that a mean dose of *S. pneumoniae* of approximately 8,791 cfu/naris (1^st^ dose), and 13,830 cfu/naris (second dose) of 23F or 44,576 cfu/naris of type 6B is sufficient to activate mucosal defence causing a sustained increase in pneumococcal specific IgG and IgA antibody to be detected in mucosal washes 6 weeks after challenge. Pneumococcal colonisation or higher doses of an attenuated strain may be required to elicit T cell and immunoglobulin mediated systemic immunity.

## Supporting Information

Figure S1 **Capsular polysaccharide IgG responses to 23F or 6B pneumococci following 23F or 6B challenge, respectively.** ELISAs were performed using 23F or 6B capsule as targets to measure specific IgG levels in serum (A), NW (B) and BAL (C). Values shown are the mean antibody concentration of triplicates in µg/ml or ng/ml as shown, pre and post 23F (*n* = 7) or 6B (*n* = 8) challenge (x-axis).
(TIF)

Figure S2 **Nasal wash total cell counts following 23F challenge.** NW total cell counts from subjects (n = 8) who were challenged with 23F pneumococcus (A, first dose and B, second dose). NW were collected before challenge (Pre) and on days 2 (A2 and B2), 4 (A4 and B4) and 7 (A7 and B7) post challenge (x-axis). y axis = cell count/ml on a log_10_ scale (bar indicates Geometric Mean). Samples with no cells were given a value of 0.1.
(TIF)

Figure S3 **Correlation between BAL anti-pneumococcal IgG levels and anti-PspA IgG concentration with total lymphocyte cell count.** Pre- and post-challenge data was pooled for correlation analyses between total lymphocyte count and anti-pneumococcal (PNC) IgG titer (A and B) or Anti-PspA IgG concentration (C and D). A significant positive correlation was observed for the 23F cohort (A and C) but not for the 6B cohort (B and D). Statistical significance was determined using a Pearson correlation test. Pearson r values and P values are indicated for each graph.
(TIF)

Table S1 **BAL differential counts (mean ± SD).**
(DOC)
