# Supplementary figures and images for: Human Nasal Challenge with Streptococcus pneumoniae Is Immunising in the Absence of Carriage
Source: PLoS Pathog. 2012 Apr 5;8(4):e1002622. doi: 10.1371/journal.ppat.1002622 (PMC3320601; doi:10.1371/journal.ppat.1002622)

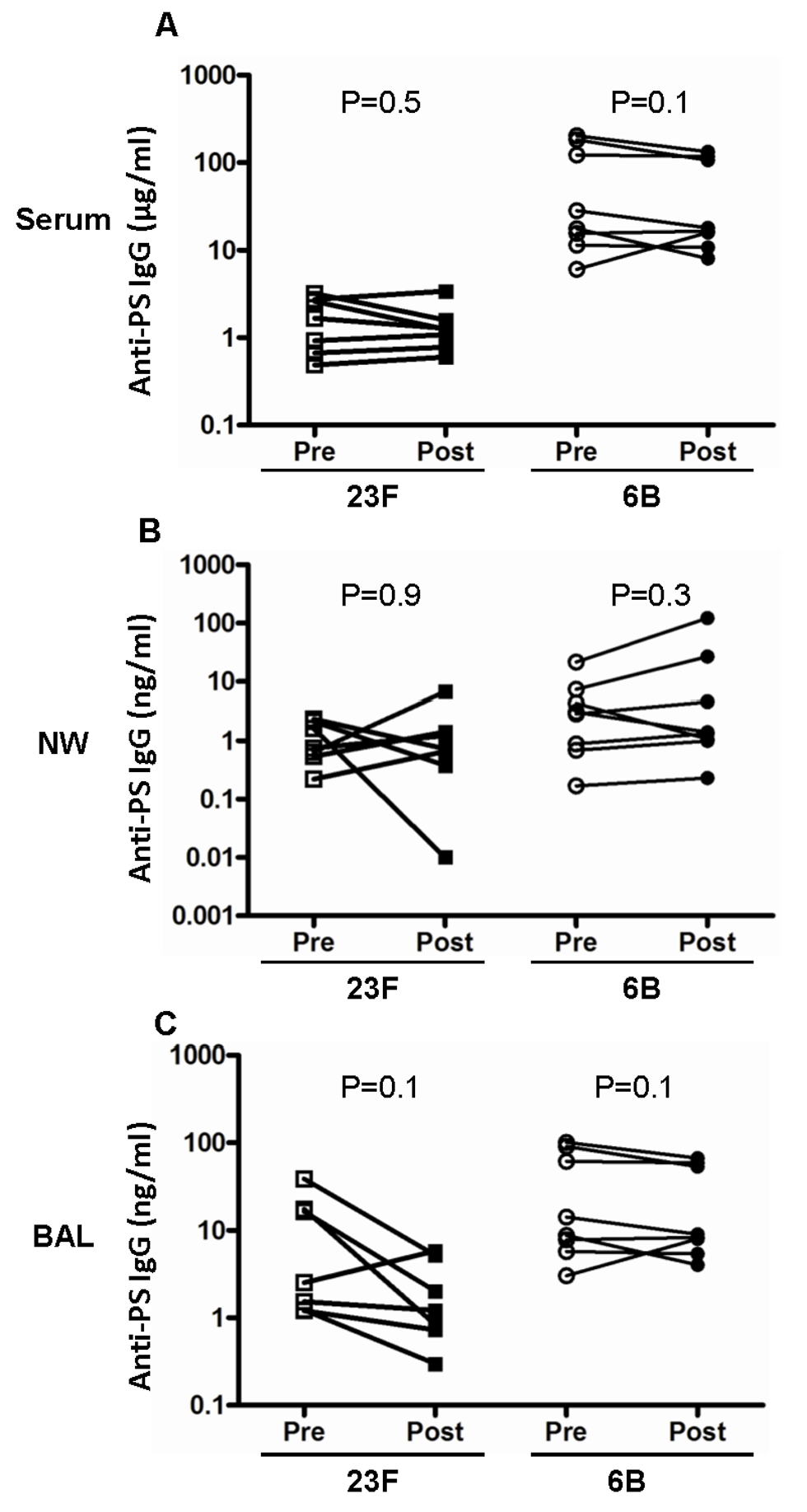

Supplement: Figure S1 — Capsular polysaccharide IgG responses to 23F or 6B pneumococci following 23F or 6B challenge, respectively. ELISAs were performed using 23F or 6B capsule as targets to measure specific IgG levels in serum (A), NW (B) and BAL (C). Values shown are the mean antibody concentration of triplicates in µg/ml or ng/ml as shown, pre and post 23F (n = 7) or 6B (n = 8) challenge (x-axis). (TIF) [file ppat.1002622.s001.tif]

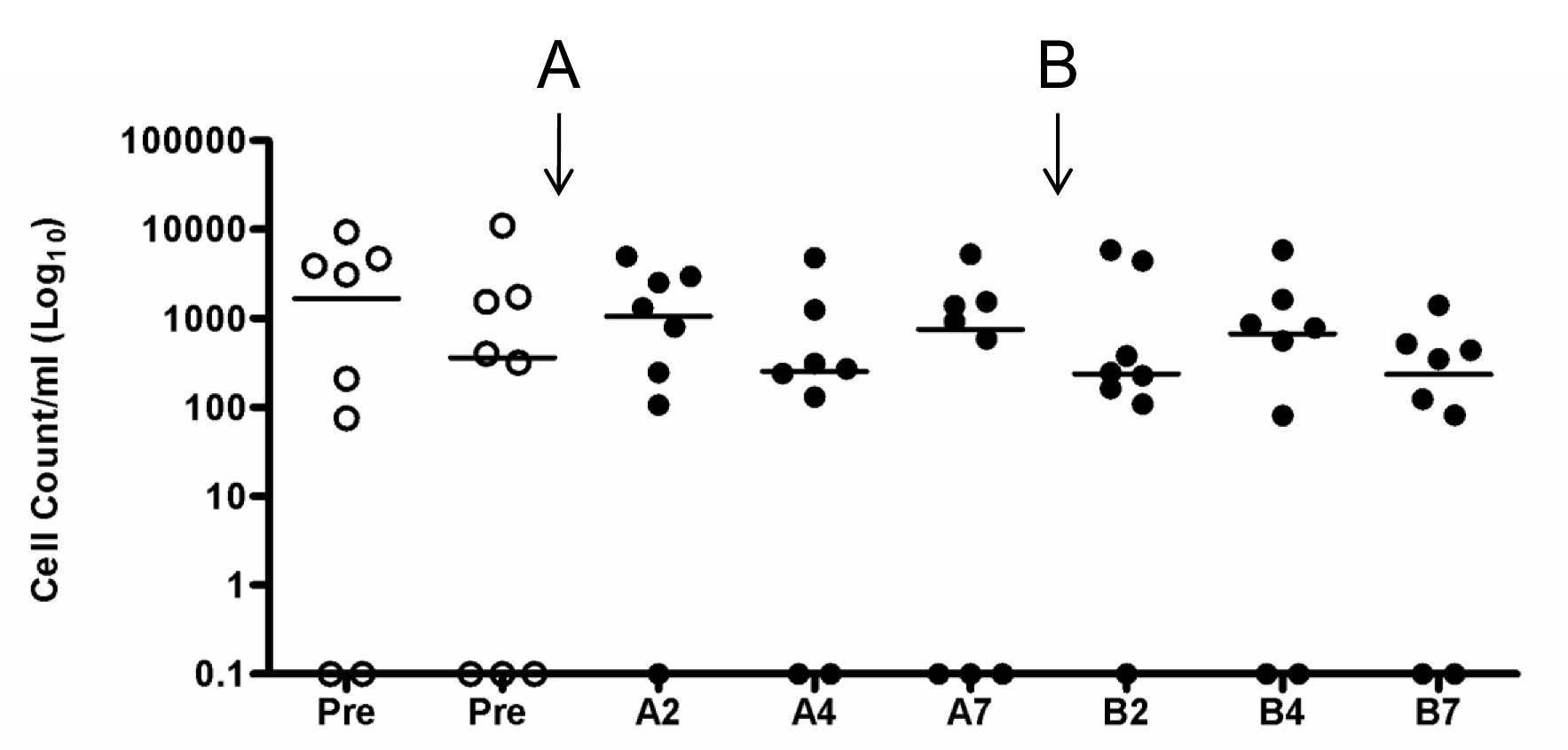

Supplement: Figure S2 — Nasal wash total cell counts following 23F challenge. NW total cell counts from subjects (n = 8) who were challenged with 23F pneumococcus (A, first dose and B, second dose). NW were collected before challenge (Pre) and on days 2 (A2 and B2), 4 (A4 and B4) and 7 (A7 and B7) post challenge (x-axis). y axis = cell count/ml on a log10 scale (bar indicates Geometric Mean). Samples with no cells were given a value of 0.1. (TIF) [file ppat.1002622.s002.tif]

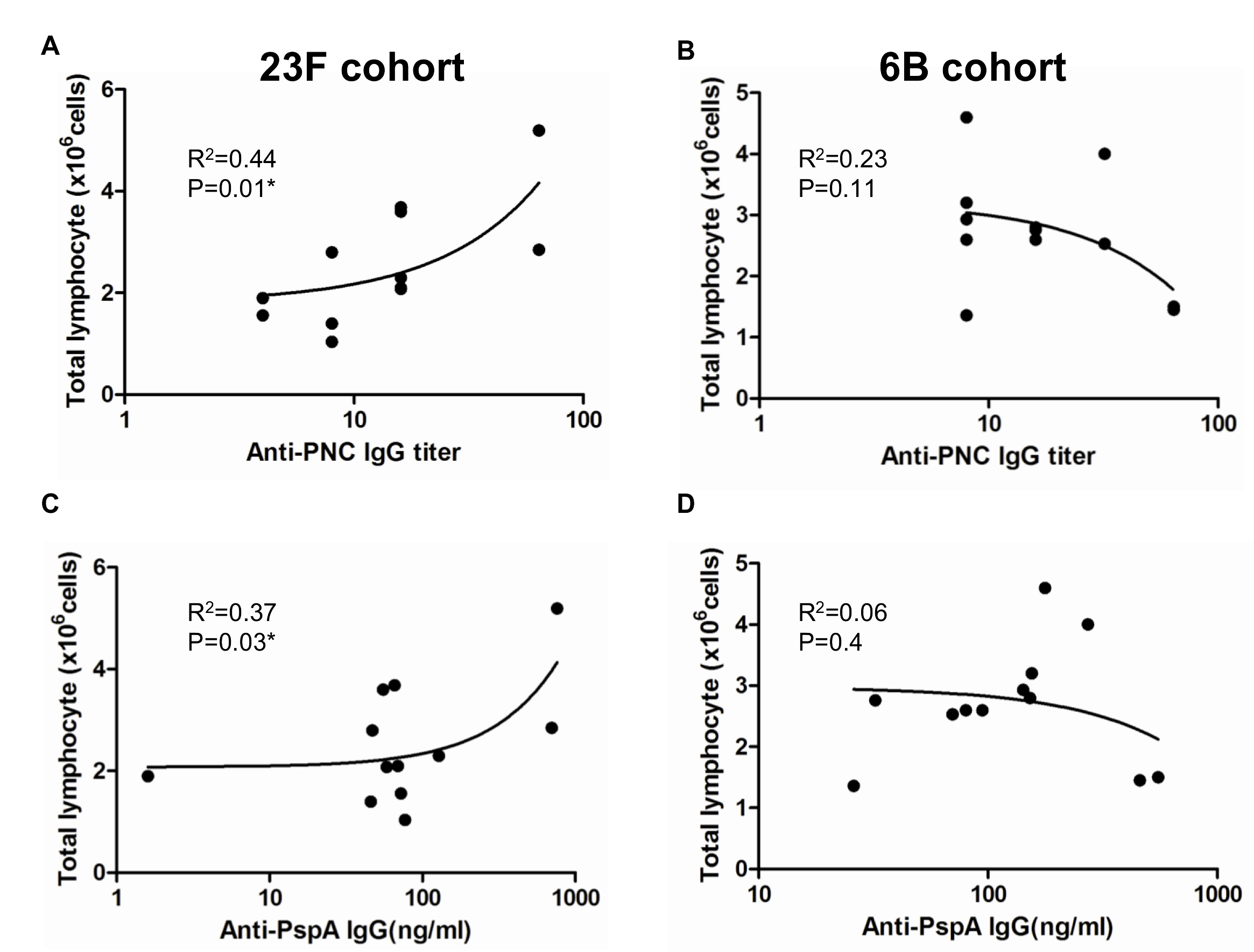

Supplement: Figure S3 — Correlation between BAL anti-pneumococcal IgG levels and anti-PspA IgG concentration with total lymphocyte cell count. Pre- and post-challenge data was pooled for correlation analyses between total lymphocyte count and anti-pneumococcal (PNC) IgG titer (A and B) or Anti-PspA IgG concentration (C and D). A significant positive correlation was observed for the 23F cohort (A and C) but not for the 6B cohort (B and D). Statistical significance was determined using a Pearson correlation test. Pearson r values and P values are indicated for each graph. (TIF) [file ppat.1002622.s003.tif]
