# Supplementary material for: Human Nasal Challenge with Streptococcus pneumoniae Is Immunising in the Absence of Carriage
Source: PLoS Pathog. 2012 Apr 5;8(4):e1002622. doi: 10.1371/journal.ppat.1002622 (PMC3320601; doi:10.1371/journal.ppat.1002622)
Supplement: Table S1 — BAL differential counts (mean ± SD). (DOC) [file ppat.1002622.s004.doc]

Table S1. BAL differential counts (mean ± SD)

|  | 23F | | 6B | |
| --- | --- | --- | --- | --- |
|  | Pre (*n*=8) | Post (*n*=8) | Pre (*n*=9) | Post (*n*=10) |
| Volume returned (ml) | 96 ± 46 | 117 ± 22 | 116 ± 22 | 120 ± 25 |
| Total cells (x10^6^/ml) | 0.1 ± 0.07 | 0.1 ± 0.05 | 0.42 ± 1.0 | 0.09 ± 0.04 |
| Macrophages (%) | 91.2 ± 5.8 (*n*=7) | 92.8 ± 3.9 (*n*=7) | 93.36 ± 2.7 (*n*=8) | 95.4 ± 2.4 (*n*=9) |
| Lymphocyte (%) | 7.1 ± 6.5 | 5.8 ± 3.9 | 5.0 ± 2.1 | 3.4 ± 2.4 |
| Neutrophil (%) | 1.6 ± 1.3 | 1.5 ± 0.9 | 1.6 ± 1.6 | 1.3 ± 0.9 |
